# Axis-space framework for cable-driven soft continuum robot control via reinforcement learning

**DOI:** 10.1038/s44172-023-00110-2

**Published:** 2023-09-05

**Authors:** Dehao Wei, Jiaqi Zhou, Yinheng Zhu, Jiabin Ma, Shaohua Ma

**Affiliations:** 1 Tsinghua Shenzhen International Graduate School (SIGS), Tsinghua University, 518055 Shenzhen, China; 2 Tsinghua-Berkeley Shenzhen Institute (TBSI), 518055 Shenzhen, China

**Keywords:** Biomedical engineering, Information technology

## Abstract

Cable-driven soft continuum robots are important tools in minimally invasive surgery (MIS) to reduce the lesions, pain and risk of infection. The feasibility of employing deep reinforcement learning (DRL) for controlling cable-driven continuum robots has been investigated; however, a considerable gap between simulations and the real world exists. Here we introduce a deep reinforcement learning-based method, the Axis-Space (AS) framework, which accelerates the computational speed and improves the accuracy of robotic control by reducing sample complexity (SC) and the number of training steps. In this framework, the SC was reduced through the design of state space and action space. We demonstrate that our framework could control a cable driven soft continuum robot with four tendons per section. Compared with the Double Deep Q-learning Network (DDQN) controller, the proposed controller increased the convergence speed by more than 11-fold, and reduced the positioning error by over 10-fold. This framework provides a robust method for soft robotics control.

## Abstract (web summary)

Shaohua Ma and colleagues introduce a deep reinforcement based control framework for cable driven soft continuum robots with reduced computational time and high positioning accuracy. This work enables an easily implemented spatial partitioning technique for the control of cable-driven soft continuum robots for minimally invasive surgery.

## Introduction

Minimally invasive surgery (MIS) involves the use of surgical instruments to access desired surgical targets inside the human body through a small incision hole. In this case, MIS offers reduced risks and injuries for patients. Soft continuum robots have distal dexterity and structural compliance, which play an important role in MIS^1,2^. Many kinds of soft continuum robots have been designed for MIS^3^. At present, the control of these robots is inaccurate due to the nonlinear behaviors of the flexible manipulator, including structural deformations, interactions with soft tissues, and collisions with other instruments^4^. Many control methods have been proposed to improve the motion accuracy of continuum robots, which can be categorized into model-based and model-free methods.

For model-based control methods, the piecewise constant curvature assumption is commonly adopted in existing models of continuum robots. This allows for computationally efficient closed-form solutions for many continuum robots in an ideal environment by ignoring environmental interactions and other nonlinear factors^5^. The Cosserat rod model allows external factors to be taken into consideration but is computationally expensive for real-time control^6^. There are also other model-based methods for continuum robot control based on finite element principles^7^. However, the unknown disturbances on the robot in the soft tissue environment can often be an obstacle for accurate navigation, whereas integrating a flexible force sensor with a robot is hard to implement due to the narrow spaces and high cost. Although fiber Bragg grating optical sensors have been used to acquire the external force or shape of a continuum robot, they are limited by small tensile strength and high cost^8^.

In contrast, model-free control methods for soft continuum robots do not need prior knowledge on the underlying mechanics or dynamics of the robot^9^. Machine learning techniques, such as neutral networks, extreme learning machines, and Gaussian mixture regression, have been implemented to model soft robot controllers^10^. However, these methods suffer from poor reliability when addressing real world challenges that a robot has never encountered in training. Data-driven and empirical methods have been utilized for updating these controller models during real-time control. For example, hybrid robot controller combining inverse kinematic and PID controller was developed to compensate the positioning error caused by external disturbance^11^. Thuruthel et al. reported a machine learning-based approach for the closed-loop kinematic control of continuum manipulators in a task space^12^. However, the control performance still heavily depends on the huge training data size and sample efficiency. DRL has been applied to robot control and the gap between simulation and real world often limited the application and efficiency of DRL controller. Deep reinforcement learning (DRL) has been recently investigated for soft continuum manipulator control. Generally, DRL methods can be divided into value-based and policy-based DRL^13^. Value-based DRL algorithms, such as the deep Q-learning network (DQN)^14^, have been applied to the 3-dimensional (3D) motion of a soft continuum robot^14^. When applying DRL to robotics control, many researchers choose to pretrain on a simulator and then transfer it to the real world^4,15,16^. However, there is a gap between the simulation and real world.

Here, we introduced an easily-implemented methodology, the Axis-space (AS) framework, to expedite convergence in DRL by reducing the SC and training steps. The feasibility and augmentation of using AS framework-based DRL has been investigated on DQN and DDQN controllers for a cable driven soft continuum robot^17,18^. Here, we investigate a cable driven soft continuum robot with four tendons per section^19^, which serves as the experimental platform. More cable driven soft continuum robots can be found in Supplementary Table 1. The AS based controllers, that is, ASDQN and ASDDQN controllers, were proven to have increased performance, including largely reduced sample data size and high position tracking precision, than their non-AS counterparts under various circumstances that might be encountered during MIS.

## Results

### Formulation of ASDDQN controller

**Fig. 1:**
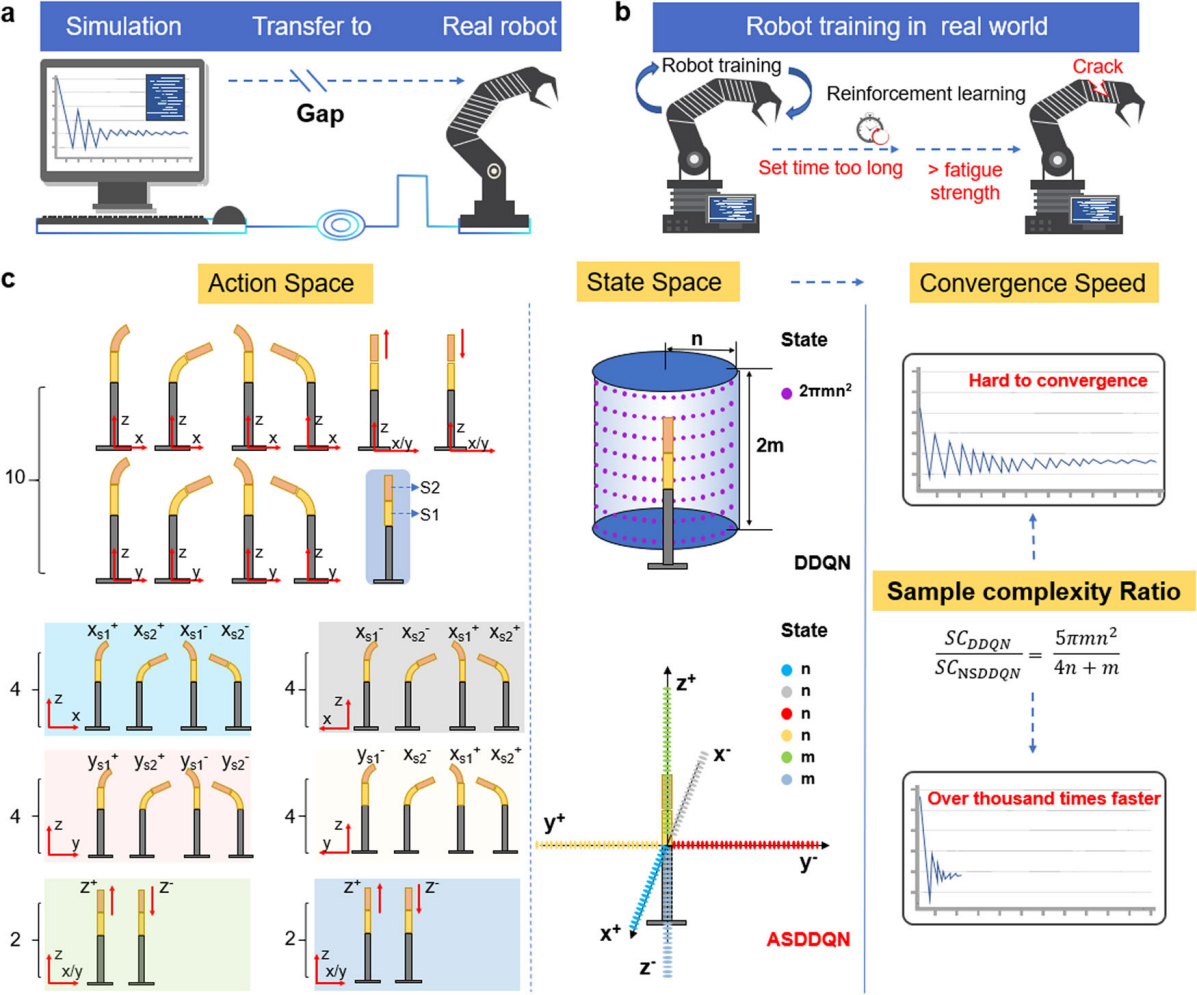
Comparison of normal value-based deep reinforcement learning algorithm (e.g., DDQN) and AS framework-based DDQN controllers, i.e., ASDDQN. **a** The gap between simulation and real-world scenarios. **b** Direct robot training in the real world poses challenges such as long setup time and the risk of robot arm damage. **c** Illustration of the design of action and state spaces for DDQN and ASDDQN controllers, and comparison of sample complexity between the two controllers. The action space of DDQN controller is 10, while that of ASDDQN controller is 2, 2, 4, 4, 4, 4 for six different sub-spaces. The state space of DDQN is $2{{{{{\rm{\pi }}}}}}{{{{{\rm{m}}}}}}{n}^{2}$, whereas that of ASDDQN controller is 4n + 2 m. A comparison of the sample complexity of DDQN and ASDDQN controllers reveals that the latter requires less sample complexity, resulting in faster convergence than the former. (*x*, *y*, *z*, *S*1, *S*2 represent *x*-axis, *y*-axis, *z*-axis, first segment, and second segment)

The gap between simulation and reality was caused by discrepancies^20^ (Fig. 1a). Although many studies have focused on the simulation-to-real world transition to narrow the reality gap in various robot control problems^21–24^, there is still a long way to go before filling this gap. The mismatch between the simulated and real-world environments raises the demand for simulation-to-real world transfer of the knowledge acquired in simulations^25^. However, this transfer would hardly work, especially when it applies to a complex robot with high degrees of freedom (DoFs), because the gap between the real environment and the simulator becomes remarkable^16^. In addition, directly implementing DRL training with robots possessing high DoFs in real world require high SC and large training episodes. The response time of robot movement resulted in a protracted training time of directly training in real world in comparison to simulation-based training. Moreover, owing to the constraints on Fatigue strength of a soft continuum robot, the robot arm fractures when the requisite training steps surpass the maximum threshold it can endure. Therefore, training robots with high DoFs using DRL in the real world faces considerable challenges (Fig. 1b).

The implementation of the AS framework, featuring a revamped state space and action space, yields reduction in SC, facilitating real-world robot training and expediting convergence rates. As shown in Fig. 1c, six subspaces, i.e., *X*+ subspace, *X*− subspace, *Y*+ subspace, *Y*− subspace, *Z*+ subspace, and *Z*− subspace, namely, subspaces 1–6, were constructed, and a different DRL model was developed for each subspace. During implementation, every target position within the robot’s 3D workspace was decomposed into vectors along the three subspaces. These vectors served as the targets for DDQN of the corresponding model. Six DDQN models in the six subspaces comprised the ASDDQN controller. In the ASDDQN model, the controller utilizes the end-tip position of the robot, obtained from the motion capture system (MCS), along with the target position as inputs to generate action signals, which are then transmitted to the soft continuum robot (Fig. 1c).

Compared with DDQN controller, AS framework-enabled DDQN controller, ASDDQN, can largely reduce sample complexity (SC) that plays an important role in training performance^26^, and its optimal *Q*-function can be estimated with *ε*-accurate in entry wise as $\frac{|S||A|}{{(1-{{{{{\rm{\gamma }}}}}})}^{3}{{{{{{\rm{\varepsilon }}}}}}}^{2}}$ for a *γ*-discounted finite-horizon MDP with state space *S* and action space *A*. In the DDQN controller, the action space ${A}_{{{{{{{\rm{DDQN}}}}}}}}$ is 10, including ${x}_{S1}^{+}$, ${x}_{S1}^{-}$, ${y}_{S1}^{+}$, ${y}_{S1}^{-}$, ${x}_{S2}^{+}$, ${x}_{S2}^{-}$, ${y}_{S2}^{+}$, ${y}_{S2}^{-}$, *z*^+^, *z*^−^, among which ${x}_{S1}^{+}$ refers to the section 1 of the robot arm bends towards the direction of ${x}^{+}$ axis. While in the ASDDQN controller, the action space of the model in ${z}^{+}$ or ${z}^{-}$ subspace (${A}_{1}$) is 2 (${z}^{+}$, ${z}^{-}$) and the action space of the model in ${x}^{+}$, ${x}^{-}$, ${y}^{+}$ or ${y}^{-}$ subspace (${A}_{2}$) is 4 (${x}_{S1}^{+}$, ${x}_{S1}^{-}$, ${x}_{S2}^{+}$, ${x}_{S2}^{-}$ or ${y}_{S1}^{+}$, ${y}_{S1}^{-}$, ${y}_{S2}^{+}$, ${y}_{S2}^{-}$). The state space ($S$) is defined as a set of states that the vectors from the real-time end tip positions of soft continuum robot to the target positions. Here, both the real-time end tip positions and the target positions can be considered as a collection of pixel dots with a diameter of 0.5 mm due to the accuracy of the MCS (NOKOV Inc., CHINA) used. Therefore, the state space contains a finite number of vectors. In work space, assuming there are 2*n* (*n* ∈ N*) states along *x* and *y* axes, 2*m* (*m* ∈ N*) states along *z* axis, there are $2\pi {{mn}}^{2}$ states totally. In our study, *n* is larger than 20 while *m* is larger than 50. The number of states in DDQN controller is ${2\pi {mn}}^{2}$, approximately $1.3* {10}^{5}$ in our study. In ASDDQN controller, the state space of the model in ${z}^{+}$ or ${z}^{-}$ subspace (${S}_{1}$) is m and the state space of the model in ${x}^{+}$, ${x}^{-}$, ${y}^{+}$ or ${y}^{-}$ subspace (${S}_{2}$) is n. Therefore, the ${{{{{{{\rm{SC}}}}}}}}_{{{{{{{\rm{DDQN}}}}}}}}$ is shown as follows:

1 $${{{{{{{\rm{SC}}}}}}}}_{{{{{{{\rm{DDQN}}}}}}}}=\frac{\left|S\right|\left|A\right|}{{\left(1-{{{{{\rm{\gamma }}}}}}\right)}^{3}{{{{{{\rm{\varepsilon }}}}}}}^{2}}=\frac{2\pi m{n}^{2}* 10}{{\left(1-{{{{{\rm{\gamma }}}}}}\right)}^{3}{{{{{{\rm{\varepsilon }}}}}}}^{2}}=\frac{20\pi m{n}^{2}}{{\left(1-{{{{{\rm{\gamma }}}}}}\right)}^{3}{{{{{{\rm{\varepsilon }}}}}}}^{2}}$$

and ${{{{{{{\rm{SC}}}}}}}}_{{{{{{{\rm{NSDDQN}}}}}}}}$ is as follows:

2 $${{{{{{{\rm{SC}}}}}}}}_{{{{{{{\rm{ASDDQN}}}}}}}}=\frac{\left|{{{{{\rm{S}}}}}}\right|\left|{{{{{\rm{A}}}}}}\right|}{{\left(1-{{{{{\rm{\gamma }}}}}}\right)}^{3}{{{{{{\rm{\varepsilon }}}}}}}^{2}}=\frac{{2* S}_{1}* {A}_{1}+4* {S}_{2}* {A}_{2}}{{\left(1-{{{{{\rm{\gamma }}}}}}\right)}^{3}{{{{{{\rm{\varepsilon }}}}}}}^{2}}=\frac{4m+16n}{{\left(1-{{{{{\rm{\gamma }}}}}}\right)}^{3}{{{{{{\rm{\varepsilon }}}}}}}^{2}}$$

According to Eqs. (1) and (2), we have

3 $$\frac{{{{{{{{\rm{SC}}}}}}}}_{{{{{{{\rm{DDQN}}}}}}}}}{{{{{{{{\rm{SC}}}}}}}}_{{{{{{{\rm{ASDDQN}}}}}}}}}=\frac{5\pi m{n}^{2}}{m+4n}$$

which in our study is approximately 2.4 × 10^3^. The results indicate that a larger work space confers greater advantages to the ASDDQN controller. The induction above indicates that the AS framework requires fewer samples to obtain the same accuracy compared with DDQN and realizes fast convergence with the small training dataset instead of high data acquisition costs and time consumption.

### The robot system design

**Fig. 2:**
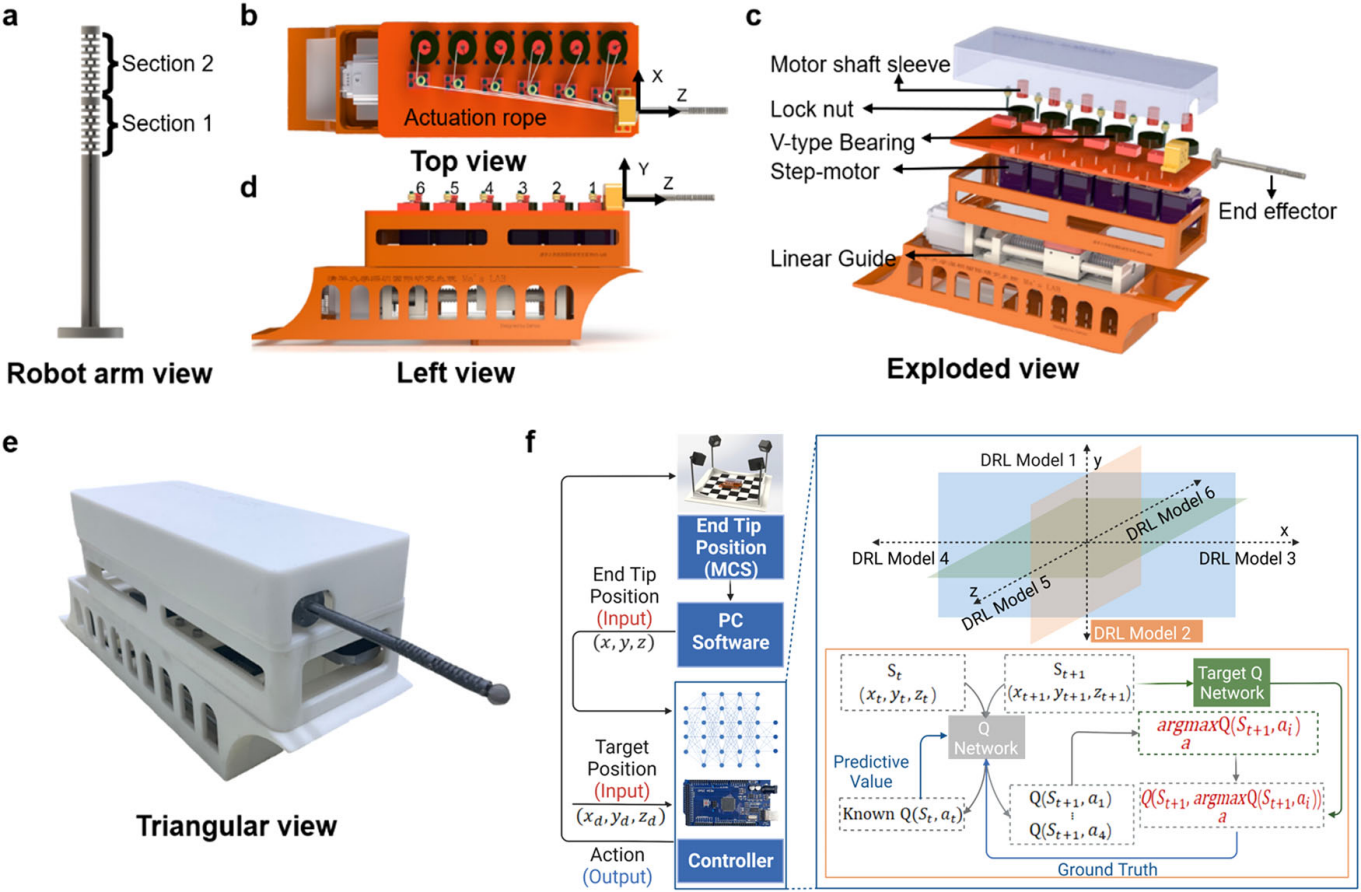
Overview of the cable-driven 5-DOF soft continuum robot and its control system. **a** The side view of the manipulator tip with two segments. **b**–**e** Multiple views of the robot: **b** top view, **c** exploded view, **d** left view, and **e** triangular view of the robot. **f** The control system consists of a motion capture system, an end robot arm, and a deep reinforcement learning controller. The tip position is obtained by the motion capture system and transferred to the deep reinforcement learning controller as the input. The controller outputs the action signals to the motor driver and controls the robot (($x,y,z$) represents the current robot arm end tip position, (${x}_{{{{{{\rm{d}}}}}}},{y}_{{{{{{\rm{d}}}}}}},{z}_{{{{{{\rm{d}}}}}}}$) represents the desired target position, (${x}_{t},{y}_{t},{z}_{t}$) represents the robot arm end tip position at time $t$, $Q({S}_{t},{a}_{t})$ represents the action-value function at time *t*).

**Fig. 3:**
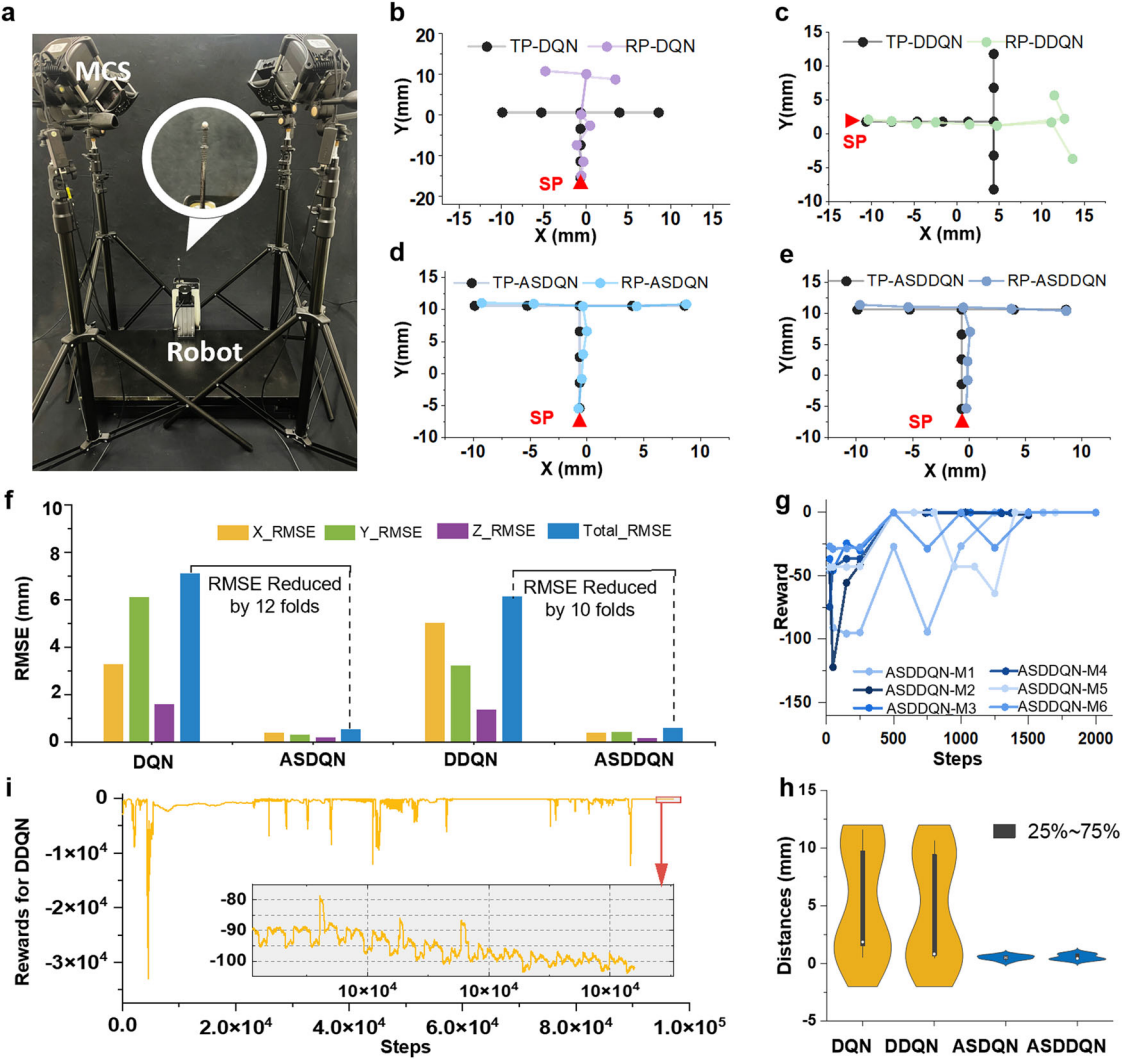
Comparison of the performance of DQN and DDQN with ASDQN and ASDDQN. **a** Experimental setup to evaluate the continuum robot performance under ASDDQN. **b**–**e** Evaluation of trajectory precision with **b** DQN (RMSE: >6.00 mm), **c** DDQN (RMSE: >6.00 mm), **d** ASDQN (RMSE: <0.61 mm), and **e** ASDDQN (RMSE: <0.61 mm) (SP start position of robot, TP target position, RP real position). **f** The trajectory tracking RMSE under the DQN, DDQN, ASDQN, and ASDDQN controllers (X_RMSE root mean square position error of *x*-axis). **g** Training performance of the ASDDQN controller within 2000 steps (M1, M2, M3, M4, M5, M6 means six models in ASDDQN controller). **h** The evaluation performance of the DQN, DDQN, ASDQN and ASDDQN controllers. **i** Training performance of the DDQN controller, which did not converge after 97,000 steps of training.

A 47.2-mm-long and 7-mm-diameter continuum surgical robot-arm allowing the active distal dexterous manipulation was developed (Fig. 2a–e). It had a central lumen with an inner diameter of 1 mm, sufficient to accommodate one instrument, such as a miniature camera, surgical forceps, and suction irrigation tubes (Supplementary Fig. 1a–c and Supplementary Table 2). It has two distal bending sections, defined as *S*1 and *S*2 (Supplementary Fig. 2), consisting of a series of 16 disks evenly distributed along the axis with two cylindrical rods connecting every two disks to function as the supporting backbone and the bending joint. Adjacent joints were arranged orthogonally to each other to achieve 2-DoF bending motion. The overall experimental setup and workflow are shown in Fig. 2f. The MCS measured the tip position of the continuum robot arm by the fiducial markers attached to the proximal and distal end of the robot (Fig. 3a). The hyperparameters used are shown in Table 1.

**Table 1:** Hyperparameters

Hyperparameter	Value
Discount factor *γ*	0.95
Replay buffer size	10,000
Learning rate *α*	0.0001
Batch size	64
Max episodes	80
Initial *ϵ*	0.4
*ϵ* decay rate	0.0001
Target network update frequency *C*	200

### “T” pattern point tracking comparison: DQN, DDQN, ASDQN, and ASDDQN

In this section, the models after training for 80 episodes, 25 steps per episode, of DQN, DDQN, ASDQN and ASDDQN were evaluated by comparing their performance under the condition that the robot could move freely in the workspace when tracking “T” patterns. The start position (red triangle) was fixed, and every subsequent step was taken toward next target position. The colored dots and curves in Fig. 3b–e represent the tracked trajectories of stepwise locomotion from the four different controllers, while the black dots represent the target positions. In Fig. 3b, in DQN controller the robot tip was positioned with high precision from the starting point to the fifth trajectory point, but from the sixth to the ninth point, it kept moving straight upwards instead of turning left or right to comply with the trajectory command. It formed a significantly deformed “T” pattern when referring to the target “T” pattern. A similar presentation is shown in Fig. 3c via the DDQN controller. The root mean square error (RMSE) of tracking dislocation was defined as the distance between the target point and the real point the robot reached. The RMSE of the DQN controller in the whole trajectory was 7.12 mm while the RMSE of the DDQN controller in the whole trajectory was 6.12 mm. These results presented the below-satisfactory performance of DQN and DDQN controllers on this robot due to limited training dataset.

The limitations of both DQN and DDQN were overcome by using the AS framework strategy. Figure 3f shows the significantly improved performance of the ASDQN and ASDDQN controllers when compared to their non-AS counterparts. The RMSE values for the whole trajectories were 7.12 and 6.12 mm under the DQN and DDQN controllers but were reduced to 0.55 and 0.61 mm under the ASDQN and ASDDQN controllers, respectively (Table 2). For both algorithms, AS-based controller achieved an over 10-fold reduction in RMSE. The obtained results demonstrated the effectiveness of the AS framework in generalization across diverse value-based DRL algorithms.

**Table 2:** Performance of DQN, DDQN, ASDQN, ASDDQN

Algorithm	Total RMSE (mm)	The average error (mm)
DQN	7.12	5.41
DDQN	6.12	4.22
ASDQN	0.55	0.52
ASDDQN	0.61	0.56

### Comparison of DDQN and ASDDQN learning performance

Real-world robot training with reinforcement learning (RL) has been challenging due to the immense amount of training data size required. To assess the efficacy of the ASDDQN controller in reducing training steps and accelerating convergence, in the training process, each AS-based model underwent 80 episodes consisting of *T* = 25 iteration steps each and the episode could be ended in advance if the distance of the target and the actual location did not exceed 0.5 mm. The rewards of the last steps in each of eight episodes were recorded, and the curve was judged as to whether they converged. Here, six neutral network models of the ASDDQN controller were selected corresponding to AS-based subspaces. The DDQN controller was tested under the same conditions. During the evaluation, the stop condition was set such that the robot had moved for 25 steps or reached the goal within an error not exceeding 0.5 mm. As shown in Fig. 3g, the reward curves of all six ASDDQN models fluctuated upwards, and eventually, all the curves converged. For the DDQN controller, the curve under the same convergence conditions showed fluctuation rather than convergence. Furthermore, under evaluation of “T” patterning, the final distance between the end tip and the target position was quantified (Fig. 3h). The tracking distance errors under the DQN and DDQN controllers were distributed in the range of 3.00–9.00 mm, while it was within 0–1.00 mm with the ASDQN and ASDDQN controllers. All the ASDDQN models converged at 60 episodes, i.e., 1500 steps, whereas the DDQN model of the DDQN controller failed to converge even after 97000 steps (Fig. 3i). The AS DDQN controller converges after 9000 training steps in total, and the convergence speed is more than ten times higher than that of the DDQN controller.

### Tracking tasks with DDQN, ASDDQN and inverse kinematic (IK) models

**Fig. 4:**
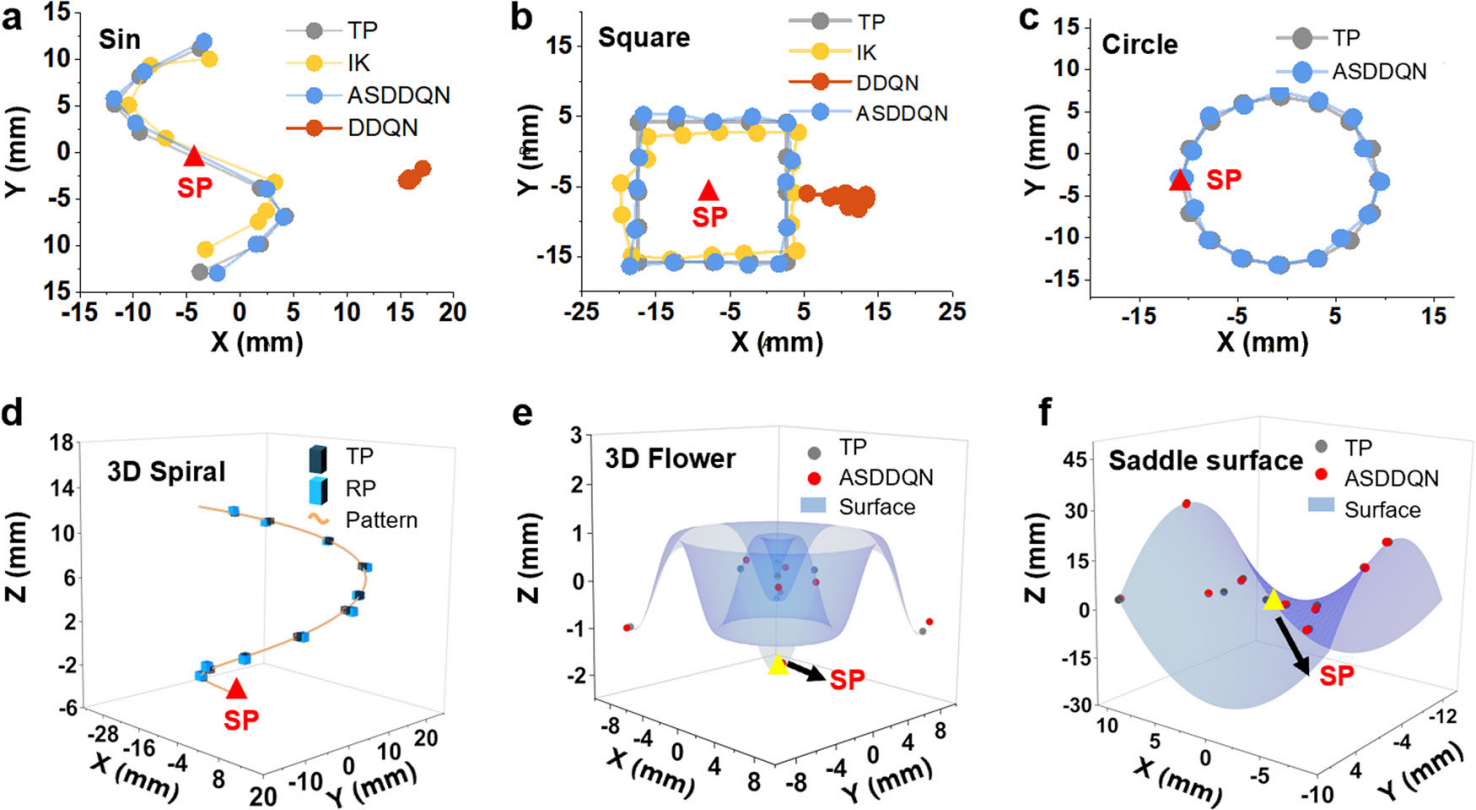
Evaluation of different controllers under complex circumstances. **a**, **b** Sin (**a**) and square (**b**) function shape tracking under ASDDQN, DDQN and IK controllers. **c**–**f** Complicated pattern tracking under the ASDDQN controller, **c** circle, **d** 3D spiral, **e** 3D flower, and **f** saddle surface (TP target position, RP real position, SP start position of robot, IK inverse kinematic).

To evaluate the performance of different controller models for more complicated tasks, the continuum robot was commanded to track two different reference trajectory patterns (sine function and square shape) in a free environment under ASDDQN and DDQN controllers. In this experiment, the robot returned to its start position every time it finished the point tracking task. An inverse kinematics model-based optimal controller was introduced. As shown in Fig. 4a, b, the RMSE values of the IK model were 2.388 mm for the square shape and 2.09 mm for the sine function. The RMSE values under the DDQN controller were 21.46 mm for the square shape and 22.29 mm for the sine function. When the ASDDQN controller was employed to perform the task of two trajectories, the RMSE values were reduced to 0.88 and 0.71 mm, respectively, an approximately 5-fold decrease from the IK model and a nearly 50-fold decrease from the DDQN controller. This proved that ASDDQN controller had dramatically improved performance in tracking complex patterns when compared with its non-AS counterpart and IK controllers.

### Irregular trajectory and complicated 3D point tracking with ASDDQN controller

In the context of a soft continuum robot executing a MIS task, it is imperative that its tip movement exhibit both precision and robustness. Such requirements are essential in ensuring the soft continuum robot can effectively respond to commands for irregular trajectories, regardless of whether it is operating in an open or tissue-enclosed environment that emulates the architecture of a biological body. To test the 3D trajectory tracking performance, the robot was commanded to track different reference shapes on two patterns that cohesively reflected the complexity of 3D movement (Fig. 4c, circle, and Fig. 4d, 3D spiral shape). The tracking RMSEs of the two patterns were 0.87 and 0.55 mm, respectively. The robot was commanded to track different reference points on two patterns that cohesively reflected the complexity of 3D movement (Fig. 4e, conical helix, and Fig. 4f, circle) to evaluate its 3D point tracking performance. The tracking RMSEs of the two patterns were 0.87 and 0.55 mm, respectively. The robot was also commanded to track reference points on a 3D sine function pattern, and the tracking RMSE was 0.65 mm (see Supplementary Fig. 3). The results showed that ASDDQN had submillimeter errors in tracking 3D shapes, which implies its significance in real surgery applications.

### Robust ASDDQN control under external payload

**Fig. 5:**
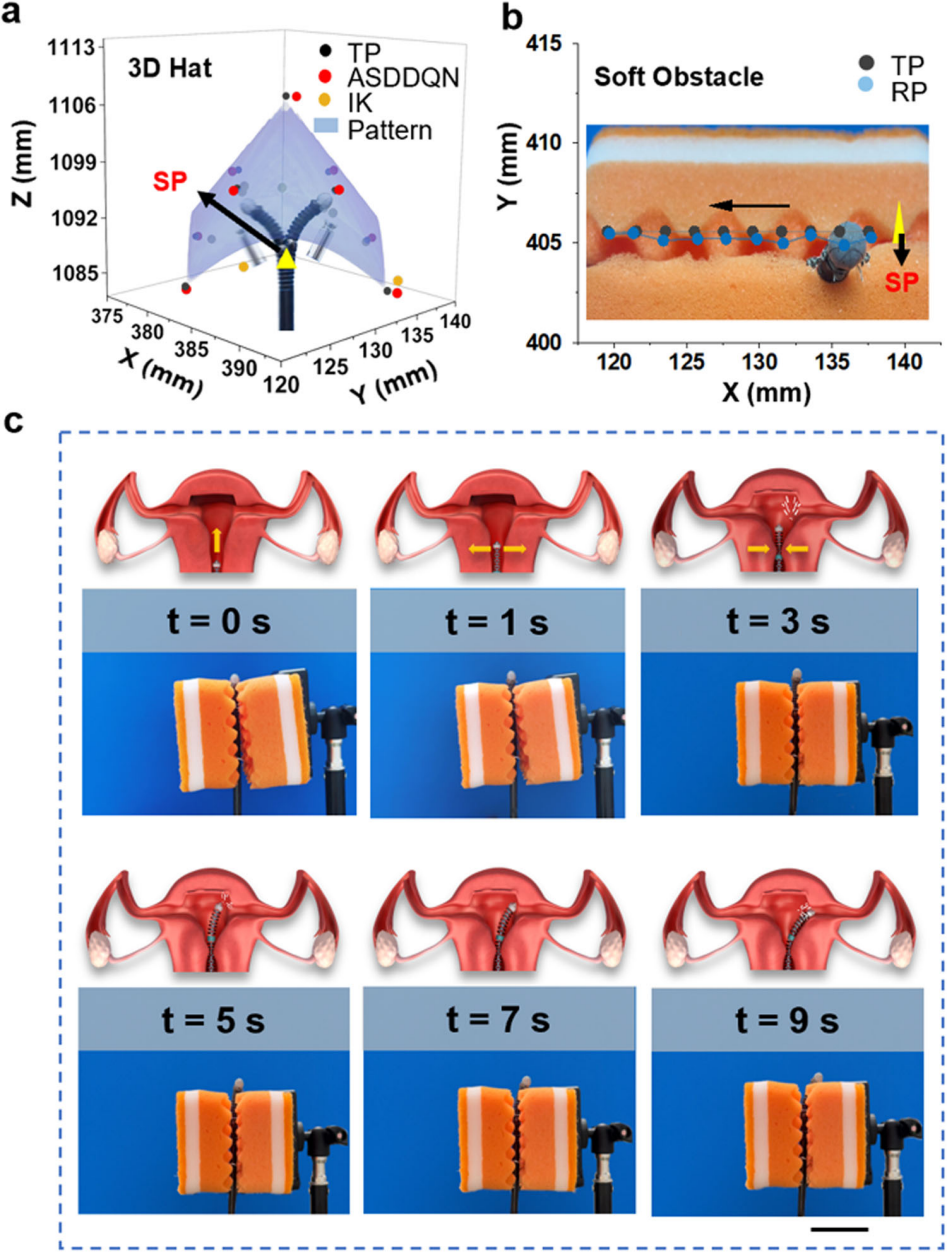
Performance evaluation in different simulation scenarios. **a** 3D hat tracking with an external payload under the ASDDQN controller. The tip was loaded with a 10 g weight. **b** Illustration of robot point tracking for endometrial repair application. **c** Time-lapse capture of real-world tracking (SP start position of robot, TP target position, RP real position, IK inverse kinematic). Scale bar, 5 cm.

To simulate the scenarios where the continuum robot performs clinical surgical operations on biological tissues or carries a load, such as an endoscopic camera or a forceps at its tip, a weight of approximately 10 g was hung at the robot arm tip (illustrated in Supplementary Movie 1). It was then commanded to track the trajectory of a 3D-hat shape. To evaluate the performance of the proposed ASDDQN controller, the IK controller was developed during point tracking and served as the comparison group. In the point tracking experiment, success was defined as the tip reaching the target position within a threshold of 0.4 mm (Fig. 5a). The performance of ASDDQN controller (RSME = 0.63 mm for the 3D-hat shape) was significantly better than that of the IK controller (RMSE = 4.85 mm for the 3D-hat shape). The inaccuracy of the IK controller in capturing robot motion under a large external payload was caused by the inverse kinematics model developed under the constant curvature assumption in capturing robot motion under a large external payload, which is not applicable in complicated scenarios where curvature becomes irregular and highly variant. The obtained results serve to demonstrate the robustness of our ASDDQN controller, a quality which can be attributed to two key factors. Firstly, it was observed that the addition of payload did not remarkably impact the performance of our robot arm. This is largely due to the inherent robustness of the arm itself, enabling it to effectively execute movements in response to instructions during operation. Furthermore, the findings also highlight the anti-interference capabilities of our ASDDQN controller, as it was observed to readily adapt its strategy when the state of the system was altered by external factors, such as the impact generated at the end of the robot arm. As a result of these robust features, the imposed load had a minimal impact on the overall performance of the system.

### The performance of ASDDQN controller in surgery simulation by using soft obstacles

The robot arm was placed between a pair of foams that served as an environmental constraint. It resembled the soft tissue analogs in the gastrointestinal wall or the inner epidermis of the uterus, aiming to investigate the online learning capability of the tracking performance in the presence of soft perturbations. The robot arm was commanded to follow a straight-line trajectory that crossed the peaks and valleys of the foam under the control of ASDDQN, which ensure the robot was in contact and pressed the soft obstacle (Fig. 5b). ASDDQN achieved performance with an RMSE of 0.49 mm, consistent with the RMSE values presented in load-free circumstances. This suggests the robustness of ASDDQN in real-world practice.

In Fig. 5c, points tracking experiment was implemented under soft obstacles (Supplementary Movie 2). After reaching final target position, the robot tip returned to its start position. The final target point was tracked 10 times, and the end tip position error was 0.53 mm. This precision level meets the requirement for most MIS surgeries^26^.

## Discussion

In this study, an AS framework was proposed to reduce the SC and speed up the convergence. The superiority of controlling the cable driven soft continuum robot using AS framework-based DRL was presented on a cable driven soft continuum robot with four tendons per section, and compared to normal DRL and a traditional inverse kinematic-based controller.

The time and computation consumption were dramatically reduced due to the reduction of SC using the user-friendly AS-based DRL methods when the convergence of the AS-based DRL controller was rapidly reached after 9000 steps compared with the circumstance that nonconvergence was reached even after 97000 steps of training in the non-AS DRL controller. It also eliminates the dependence on the virtual simulation environment in real-world DRL training of multi-DoFs for soft continuum robots.

Both algorithmic and robotic generalization were promising in the AS framework employed in this study. The implementation of the AS-based controller on diverse value-based RL algorithms, i.e., DQN and DDQN, lead to noteworthy enhancements in both convergence speed and controlling accuracy of soft robot movements. Currently, we have exclusively applied the AS framework to the cable driven soft continuum robot. However, the AS framework holds potential for broader application across other soft continuum robots, provided they possess partitionable task spaces.

The physical experiments demonstrated that AS framework-based RL consistently yielded submillimetre tracking accuracy and high stability under different test environments, including different tracking patterns and tracking under external payloads and soft obstacles. The navigation accuracy in space by AS augmentation was increased by over 10-fold when compared with counterpart DRL methods and over 5-fold versus the inverse kinematic-based methods.

Further work involves developing a more stable multi-DOF soft continuum robot that can undergo larger training episodes to meet the needs of more actual applications. Notably, there is in urgent demand to develop open-loop AS-framework-based DRL controllers that incorporate end-tip orientation for soft continuum robots capable of meeting the requirements of clinical applications. Although the soft robot demonstrated robust performance under payload or when obstructed by a soft-tissue analog, its robust performance under different real anatomic conditions remain to be examined. Eventually, it may pave the way for clinically applicable robots that hold promises to revolutionize medical operations. The motion capture implemented by MCS as in this study is transferable to medical radiography in motion capture and guidance.

## Methods

### Robot design

The robot arm was 3D-printed using stereolithography of sterilizable high-strength nylon to allow potential integration with imaging modalities, such as magnetic resonance imaging (MRI). It was driven by six Gathering wire rope (Hongxiang Company, China) via six customized motor bushings (Hongtai Company, China). Six 42-stepper motors (Markerbase Company, China) are connected with the motor bushing as the actuators. Motors 1, 2, and 5 were responsible for the pitch motion (the *x*–*z* plane). Motors 3, 4, and 6 were responsible for the yaw motion (the *y*–*z* plane). The linear guide was responsible for the *z*-axis movement (Supplementary Fig. 2, Supplementary Table 2, and Movies 3 and 4).

### Experimental platform setup

A closed-loop feedback position control of the robot was used with continuous positional data from MCS. The MCS collected the sensor data, while the control program was implemented in Arduino and Python 3. The communication between the Arduino microcontroller and computer was realized by the Transmission Control Protocol/Internet Protocol. The Arduino microcontroller received action signals, delivered the corresponding signals to control the motors, then read and sent sensor data to the computer. The computer received the sensor data as the input to the DRL algorithms implemented on PARL 2.0.3. The data were processed, and the best action of the current policy was chosen and sent to the Arduino microcontroller.

### Markov decision process (MDP)

In RL, the Markov property of Markov decision processes (MDPs) is commonly exploited by RL agents. The simplest MDP can be represented as a tuple <*S*, *A*, *P*, *R*, *γ*>, where *S*, *A*, *P*, *R*, and *γ* represent the state, action, transition model, reward, and discount factor^12^. The specific definitions of the task in our control settings were defined as follows:

- State (*S*): states were defined as (${\xi }_{1}$, ${\xi }_{2}$, ${\xi }_{3}$) = $({x}_{{{{{{{\rm{target}}}}}}}}-{x}_{{{{{{{\rm{tip}}}}}}}},{y}_{{{{{{{\rm{target}}}}}}}}-{y}_{{{{{{{\rm{tip}}}}}}}},{z}_{{{{{{{\rm{target}}}}}}}}-{z}_{{{{{{{\rm{tip}}}}}}}})$, where ${\xi }_{1}$, ${\xi }_{2}$ and ${\xi }_{3}$ are the vectors between the robot’s tip position and the target position.
- Action (*A*): a total of 10 actions were defined. Actions 1–4 were responsible for the bending along *X*+ and *X*− axis, actions 5–8 for *Y*+ and *Y*−, actions 9–10 for *Z*+ and *Z*−. Each action was implemented by a specific displacement of a cable connecting to the motor. Supplementary Fig. 2 shows the correspondence between each action and cable. Actions 9 and 10 represented the linear guide moving upwards and downwards by a specific displacement, respectively. The settings for actions 1–10 in this study were adjusted according to the parameters of robots, e.g., the minimum step of a stepping motor. At each iteration, one agent selected an action ${{{{{\boldsymbol{a}}}}}}\in A$, where ***a*** represented the cable displacement in mm and a predefined action set $A=\left\{{a}_{1},{a}_{2},{a}_{3},{a}_{4},{a}_{5},{a}_{6},{a}_{7},{a}_{8},{a}_{9},{a}_{10}\right\}$.
- Reward (*R*): the reward function provided a scalar numerical signal as feedback to the agents. The reward function is defined as follows:

4 $${{{{{\rm{dist}}}}}}\left[i\right]=\sqrt{{({p}_{{{{{{{\rm{target}}}}}}}}\left[i\right]-{p}_{{{{{{{\rm{tip}}}}}}}}\left[i\right])}^{2}}$$

5 $$r=-{{{{{\rm{dist}}}}}}\left[i\right]* {e}^{{{{{{{\rm{dist}}}}}}}[i]}$$

where *i* = 0, 1, 2, 3, 4, and 5 represent spaces 1–6, respectively. Equation (4) represented the spatial Euclidean distance between the target and actual position of the end tip of a manipulator. Equation (5) ensured that the reward would be 0 if the manipulator tip reached the target position, and the continuity of the reward was also guaranteed. Soft robots were made of materials with a limit for Fatigue strength, such as nylon (fatigue strength, 12,400 psi) in this study. In real-world robot training with DRL, the robot would fracture if bending was repeated beyond the specific limit. Equation (5) was set as an exponential function with the index number being the spatial Euclidean distance between the target position and actual end tip position of the manipulator. This provided a considerable punishment when the robot was far away from the target position.

### Supplementary information

Supplemental Information

Description of Additional Supplementary Files

Supplemental Video 1

Supplemental Video 2

Supplemental Video 3

Supplemental Video 4

## Data Availability

The data that support the findings of this study are available from the corresponding author upon reasonable request.

## Code availability

Code can be accessed on GitHub at https://github.com/shaddock-123/Axis-space-AS. To utilize the code, it is necessary to first download the SEEKER software in order to obtain the position information of the optical marker.
