# Supplementary material for: Axis-space framework for cable-driven soft continuum robot control via reinforcement learning
Source: Commun Eng. 2023 Sep 5;2:61. doi: 10.1038/s44172-023-00110-2 (PMC10956126; doi:10.1038/s44172-023-00110-2)
Supplement: Supplementary file 1 — Supplemental Information [file 44172_2023_110_MOESM1_ESM.pdf]

Supplemental Materials

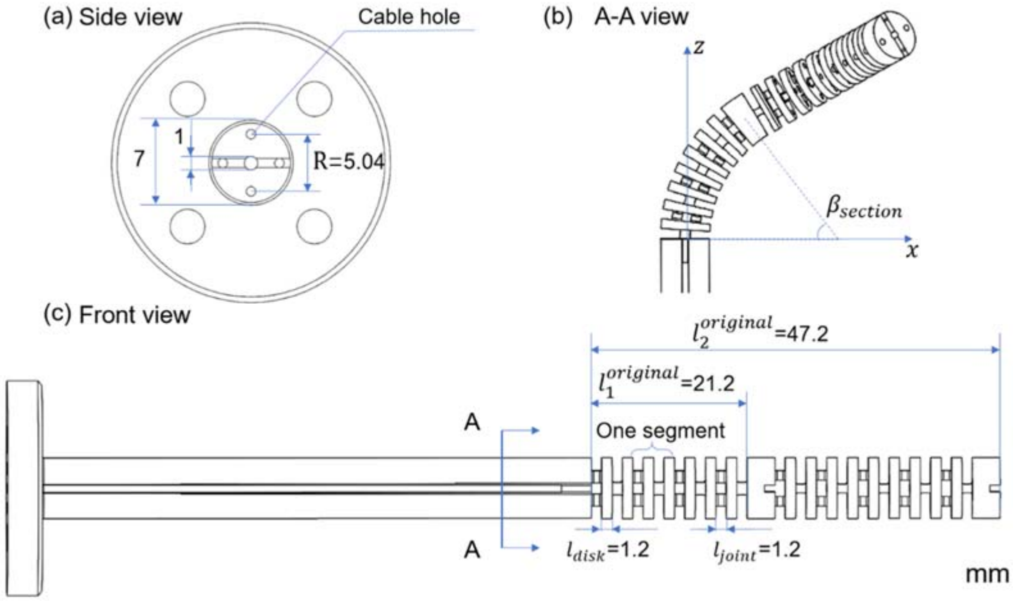

Figure S1. Robot arm overview. (a) Side view of the robot arm, the distance between the two cable holes is 5.04mm.(b) Bending status of the robot arm. (c) Front view of the robot arm.

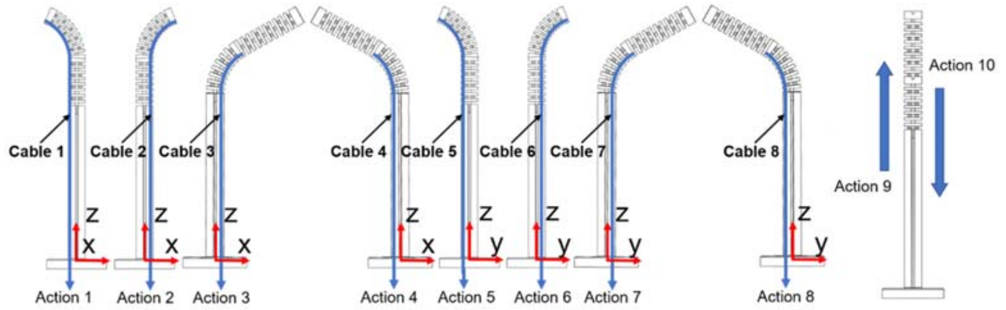

Figure S2. The relationship between the cables and actions. Action 1 represent cable 1 is driven with length change 0.16 mm. So do action 2-8. Action 9 and Action 10 represented the linear guide move upward and downward with 0.07 mm, respectively.

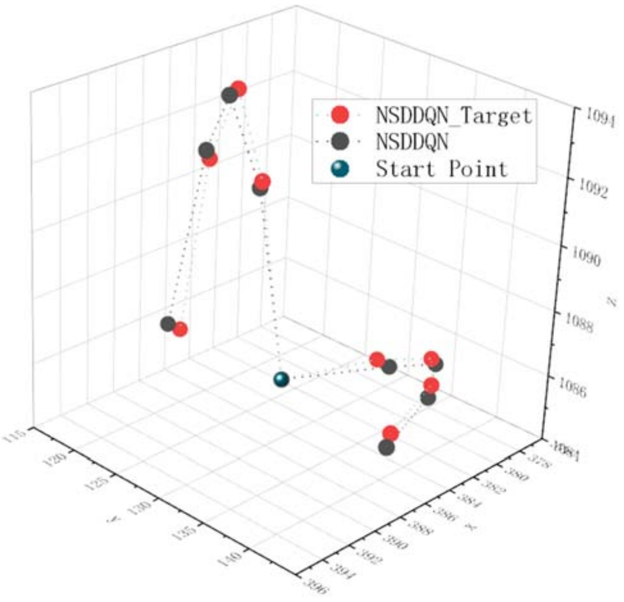

Figure S3. Point Tracking (3D sine function shape) with NSDDQN controller (Start Point at x=386mm, y=126mm, z=1086mm

Table S1. More cable driven soft continuum robots with four tendons per section

Picture	Tendon number per section	Flexible/ soft continuum robot	Cable driven or not	ref
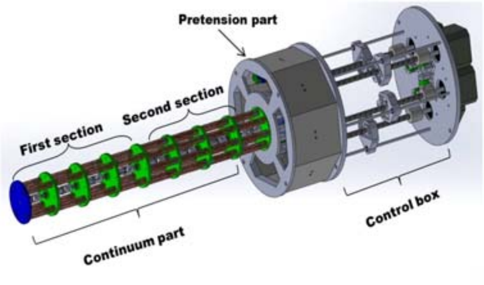	4	yes	yes	[1]
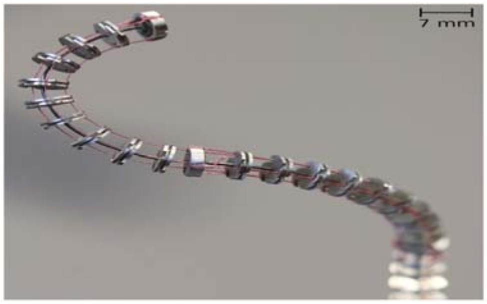	4	yes	yes	[2]
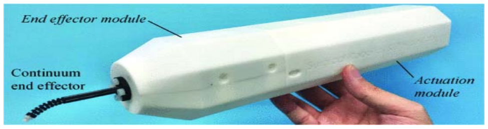	4	yes	yes	[3]
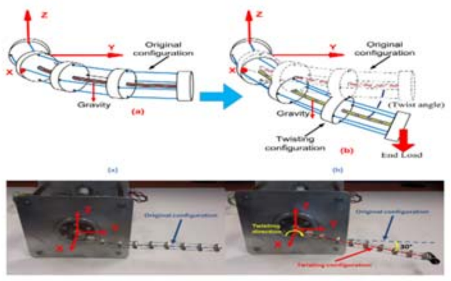	4	yes	yes	[4]
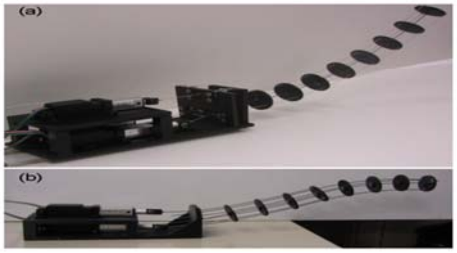	4	yes	yes	[5]

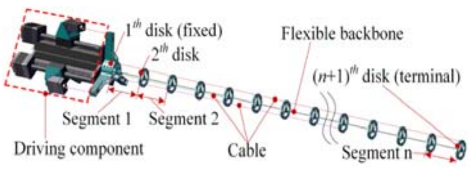	4	yes	yes	[6]
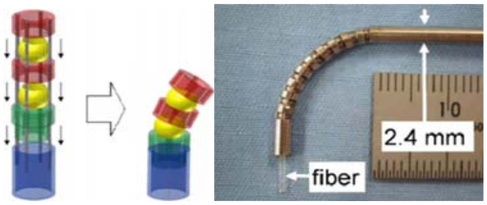	4	yes	yes	[7]
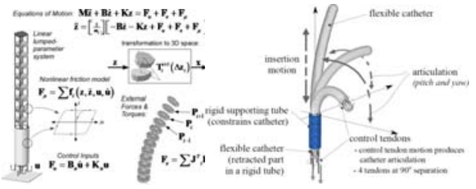	4	yes	yes	[8]
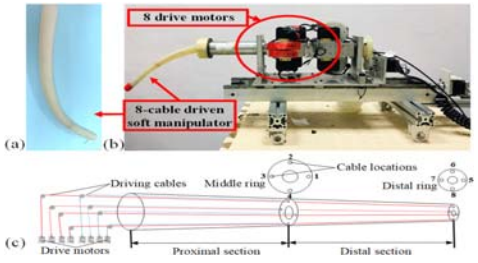	4	yes	yes	[9]
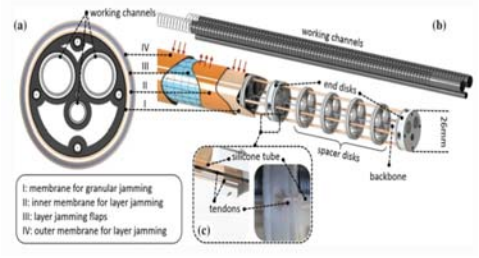	4	yes	yes	[10]

Table S2. Robot Parameters

l_{disk}	1.2mm
l_{joint}	1.2mm
$l_1^{original}$	21.2mm
$l_2^{original}$	47.2mm
N_1	4
N_2	5
R	2.52mm

where l_{disk} is the thickness of the disks, l_{joint} is the length of the compliant joints, $l_1^{original}$ is the original length of one pair of cables in first section, $l_2^{original}$ is length of one pair of cables in second section, N_1 is the quantity of segments in first single section, N_2 is the quantity of segment in second section, R is the distance from center of the disk to the cable anchor point, beta_segment is the bending angle of segment and beta_section is the bending angle of section.

Supplemental Movie 1. Point tracking of robot arm with external payload (10 g)

Supplemental Movie 2. The robot performs point tracking between the soft obstacle (side view)

Supplemental Movie 3. Explanation for cable driven control

Supplemental Movie 4. The robot movement demonstration

Supplementary Reference

1. Amanov E, Nguyen T-D, Burgner-Kahrs J. Tendon-driven continuum robots with extensible sections—A model-based evaluation of path-following motions. The International Journal of Robotics Research. 2021;40(1):7-23.

2. Q. Ding, Y. Lu, A. Kyme and S. S. Cheng, "Towards a Multi-imager Compatible Continuum Robot with Improved Dynamics Driven by Modular SMA," 2021 IEEE International Conference on Robotics and Automation (ICRA), Xi'an, China, 2021, pp. 11930-11937

3. Dong, Xin, et al. “A Novel Continuum Robot Using Twin-Pivot Compliant Joints: Design, Modeling, and Validation.” Journal of Mechanisms and Robotics, vol. 8, no. 2

4. W. S. Rone and P. Ben-Tsvi, “Continuum robotic tail loading analysis for mobile robot stabilization and maneuvering,” in Proceedings of the ASME International Design Engineering Technical Conferences & Computers and Information in Engineering Conference, pp. 1–8, Buffalo, NY, USA, August 2014.

5. Z. Liu, Z. Cai, H. Peng, X. Zhang and Z. Wu, "Morphology and Tension Perception of Cable-Driven Continuum Robots," in IEEE/ASME Transactions on Mechatronics, vol. 28, no. 1, pp. 314-325, Feb. 2023

6. K. Harada, Z. Bo, S. Enosawa, T. Chiba and M. G. Fujie, "Bending Laser Manipulator for Intrauterine Surgery and Viscoelastic Model of Fetal Rat Tissue," Proceedings 2007 IEEE International Conference on Robotics and Automation, Rome, Italy, 2007, pp. 611-616

7. J. Jung, R. S. Penning, N. J. Ferrier and M. R. Zinn, "A modeling approach for continuum robotic manipulators: Effects of nonlinear internal device friction," 2011 IEEE/RSJ International Conference on Intelligent Robots and Systems, San Francisco, CA, USA, 2011, pp. 5139-5146

8. F. Xu, H. Wang, W. Chen and Y. Miao, "Visual Servoing of a Cable-Driven Soft Robot Manipulator With Shape Feature," in IEEE Robotics and Automation Letters, vol. 6, no. 3, pp. 4281-4288, July 2021.

9. Amanov, E., Nguyen, TD., Markmann, S. et al. Toward a Flexible Variable Stiffness

Endoport for Single-Site Partial Nephrectomy. Ann Biomed Eng 46, 1498–1510 (2018).

10. Xiaohua Hu, Ang Chen, Yigang Luo, Chris Zhang & Edwin Zhang (2018) Steerable catheters for minimally invasive surgery: a review and future directions, Computer Assisted Surgery, 23:1, 21-41
