# Supplementary material for: Axis-space framework for cable-driven soft continuum robot control via reinforcement learning
Source: Commun Eng. 2023 Sep 5;2:61. doi: 10.1038/s44172-023-00110-2 (PMC10956126; doi:10.1038/s44172-023-00110-2)
Supplement: Supplementary file 2 — Description of Additional Supplementary Files [file 44172_2023_110_MOESM2_ESM.pdf]

Description of Additional Supplementary Files

File name: Supplementary Movie 1

Description: Point tracking of robot arm with external payload (10 g)

File name: Supplementary Movie 2

Description: The robot performs point tracking between the soft obstacle (side view)

File name: Supplementary Movie 3

Description: Explanation for cable driven control

File name: Supplementary Movie 4

Description: The robot movement demonstration
